# Effects of Feeding Periods of High Cholesterol and Saturated Fat Diet on Blood Biochemistry and Hydroxyproline Fractions in Rabbits

**DOI:** 10.4137/bbi.s445

**Published:** 2008-04-22

**Authors:** Mohamed A.K. Abdelhalim, N.J. Siiddiqi, A.S. ALhomida, Mohammed S. Al-Ayed

**Affiliations:** 1 King Saud University, College of Science, Department of Physics and Astronomy, Biomedical Science Group, Riyadh, Saudi Arabia; 2 King Saud University, College of Science, Department of Biochemistry, Riyadh, Saudi Arabia

**Keywords:** cholesterol, hydroxyproline, rabbits, feeding periods, atherosclerosis

## Abstract

Hypercholesterolemia and hypertriglyceridemia are considered as important risk factors during the atherosclerotic process. The aim of the present investigation was to study the total cholesterol (TC), low-density lipoprotein cholesterol (LDLC), high density lipoprotein (HDL), triglyceride (TG), platelet levels and hydroxyproline fractions during the pathogenesis of atherosclerosis. For this purpose, twenty five 12-weeks, New Zealand white male rabbits, were purchased, individually caged, and divided into either control group or cholesterol-fed group. The control group (n = 10) was fed 100 g/day of normal diet, ORC-4 (Oriental Yeast Co. Ltd., Tokyo, Japan) for a period of 15 weeks. The cholesterol-fed group (n = 15) was fed a high cholesterol and saturated fat diet of ORC-4 containing 1% cholesterol plus 1% olive oil (100 g/day) for periods of 5 (group 1), 10 (group 2) and 15 (group 3) weeks. Blood sample from each animal was taken at the end of the experimental period for the biochemical analysis. The results of the present study showed that TC, LDLC, TG, HDLC and platelets were significantly (P < 0.01) increased in cholesterol-fed rabbits as compared with control rabbits. The serum hydroxyproline (Hyp) in rabbits belonging to group 1 showed no significant alteration when compared to control group. Group 2 rabbits showed a significant increase of 103% (P < 0.01) and 100% (P < 0.001) in free and protein—bound hydroxyproline fractions respectively when compared to control rabbits. However, there was no significant change in peptide—bound and total serum hydroxyproline levels as compared to the control group (P > 0.05). There was no significant (P > 0.05) decrease of free serum hydroxyproline in group 3 rabbits when compared to control rabbits. On the other hand, group 3 rabbits showed a significant increase in peptide–bound and protein-bound Hyp by 517% (P < 0.05) and 100% (P < 0.01) respectively when compared to control rabbits. However, total serum Hyp in group 3 rabbits showed no significant (P > 0.05) change when compared to control rabbits. These results suggest that feeding rabbits high cholesterol and saturated fat diet for feeding periods of 5, 10 and 15 weeks induced significant change in TC, LDLC, HDL, TG, platelet levels and various Hyp fractions in serum without any significant change in the total Hyp content.

## Introduction

Collagens are members of a family of connective tissue macromolecules which are diverse structurally and functionally. They have been proteins of considerable interest to clinicians, biochemists, geneticists and cell biologists because of their involvement in many pathological processes. Connective tissue derives its prominent features such as mechanical strength from collagen. Hydroxyproline is a post translational product of proline hydroxylation catalyzed by the enzyme prolylhydroxylase (EC 1.14.11.2) (Pihlajaneimi et al. 1991). The occurrence of this amino acid is thought to be confined exclusively to collagen, where it is present in the Y position of the Gly-X-Y repeating tripeptide (Nemethy and Scheraga, 1986). Consequently, the presence of Hyp in tissues or serum can be used as a measure of collagen or collagen degradation products (Reddy and Enwemeka, 1996). In our previous studies, we have reported the concentrations of various Hyp fractions in the plasma (Siiddiqi and Alhomida, 2001; Siiddiqi et al. 2002), erythrocytes (Siiddiqi and Alhomida, 2002) and tissues (Siiddiqi, 2000; Siiddiqi et al. 2000; Siiddiqi et al. 2001; Siiddiqi and Alhomida, 2003) of different mammals. Collagen makes up about 50% of the dry weight of the heart and is mainly type I and type III (fibrillar type) (Mayne R, 1982). Normally the collagen breakdown occurs at a very slow and controlled rate. However, during diseases there is rapid and extensive breakdown of collagen. The final breakdown products of collagen include free and peptide—bound hydroxyproline which are not reutilized for collagen biosynthesis (Reddy et al. 1991). Protein-bound hyp on the other hand does not mirror collagen turnover but it is related to complement metabolism (Nagelschmidt and Struck, 1977).

The changes in TC, LDLC, TG, HDLC, platelet and serum hydroxyproline fractions in rabbits fed high cholesterol and saturated fat diet could provide an insight into the pathogenesis of atherosclerosis. Gregory has postulated that serum hypercholesterolemia accelerates atherogenesis by augmenting cholesterol accumulation in the arterial intima (1999). High TC and LDLC have been correlated with the increased risk of atherosclerosis (Martin et al. 1986). Triglyceride-rich lipoproteins of both intestinal and liver origin are considered to be atherogenic factors (Philips et al. 1993; Zilversmit, 1995). Hypertriglycerideia has associated with an increased risk of coronary heart disease (Hokanson and Austin, 1996; Miller et al. 1998). Thus, in the present study an attempt was made to study the serum TC, LDLC, HDLC, TG, platelet counts and Hyp concentration in rabbits fed on high cholesterol and saturated fat diet for feeding periods of 5, 10 and 15 weeks.

## Materials and Methods

### Animals

Twenty five 12-Weeks old, New Zealand white male rabbits, were purchased from Kitayama Lab. Ltd., Kyoto, Japan, individually caged, and divided into either control group or cholesterol-fed group. The control group (n = 10) was fed on 100 g/day of normal diet, ORC-4 (Oriental Yeast Co. Ltd., Tokyo, Japan) for a period of 15 weeks. The cholesterol-fed groups (the experimental groups; n = 15) were fed on high cholesterol and saturated fat diet of ORC-4 containing 1% cholesterol plus 1% olive oil (100 g/day) for feeding periods of 5 weeks (group 1), 10 weeks (group 2) and 15 weeks (group 3).

### Collection of blood and preparation of serum

Blood samples of 2 ml were obtained from the rabbits via venepuncture of an antecubital vein. Blood was collected into two polypropylene tubes viz., one for serum and one for plasma. The blood for plasma was collected in heparin. Serum was prepared by allowing the blood to clot at 37 °C and to centrifuge at 3000 rpm for ten minutes.

### Determination of total cholesterol (TC), low-density lipoprotein cholesterol (LDLC), high-density lipoprotein cholesterol (HDLC), triglyceride (TG) and platelet counts

Serum TC and TG levels were analyzed by the clinical laboratory centre of King Khaled Hospital. LDLC and HDLC concentrations were determined by the previously reported method (Lee et al. 1998 and Koenig et al. 1992). Platelet counts were measured with ADVIA 120 Haematology System (Bayer Medical, Tarrytown, N.Y.).

### Extraction of free, peptide-bound and protein-bound hydroxyproline

Free and protein-bound hydroxyproline were extracted by the method of Varghese et al., 1981 with a slight modification as described by Siiddiqi and Alhomida (2002). 0.5 ml of the plasma was treated with 2 ml of re-rectified absolute alcohol and centrifuged at 600 g for 10 min. This was repeated three times. The supernatants (which contained free and peptide-bound Hyp) (Varghese et al. 1981) were pooled and kept at 40 °C till the evaporation of ethanol. The residue was dissolved in 0.5 ml of distilled water and 50 μl of the extract was used for estimation of free hydroxyproline. The peptide-bound hydroxyproline was determined after alkaline hydrolysis of the ethanol extractable fraction. The pellets of all the samples were dissolved in an aliquot of distilled water and 50 μl of the extract was used for determination of protein-bound hydroxyproline. The precipitate obtained upon ethanol treatment of the plasma was subjected to alkali hydrolysis to determine protein-bound hydroxyproline.

### Determination of hydroxyproline concentration

Hydroxyproline was measured by the modified alkaline hydrolysis method of Reddy and Enwemeka, 1981. Briefly to 50 μl of homogenate sample was added NaOH (2 N final concentration) and the mixture was hydrolyzed by heating in boiling water bath for about 3–4 h. Approximately 900 μl 56 mM chloramines T reagent was added to the hydrolyzed sample and oxidation was allowed to proceed at the room temperature for 25 minutes. Then 1.0 ml 1 M Ehrlich’s reagent (P-dimethylaminobenzaldehyde) was added to the oxidized sample and the chromophore was developed by incubating the samples at 65 °C for 20 min. The absorbance was read at 550 nm. The hydroxyproline concentration in the samples was calculated from the standard curve of hydroxyproline. More details about the optimization, linearity, specificity, precision and reproducibility have been previously reported (Siiddiqi et al. 2000).

### Statistical analyses

The results of the present study were expressed as mean ± SD. To assess the significant differences between the control group and the cholesterol-fed group of rabbits for blood biochemistry parameters, statistical analysis using One-Way Analysis of Variance (ANOVA) for repeated measurements with the significance assessed at 5% confidence level was performed. To assess the significant differences between control group and cholesterol-fed group of rabbits for hydroxyproline fractions, Tukey’s multiple comparison test (*P < 0.05 as compared to control group; **P < 0.01 as compared to control group; ***P < 0.001 as compared to control group) with the significance assessed at 5%, 1% and 0.1% confidence levels were performed.

## Results

**Figure 1:**
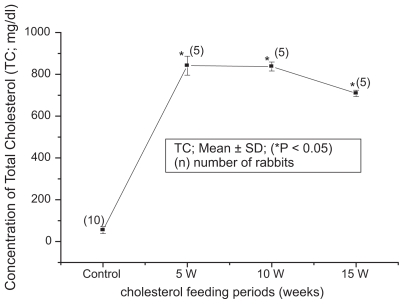
Represents TC (mg/dl) concentration in control and cholesterol-fed rabbits versus cholesterol feeding periods of 5, 10 and 15 weeks (*P < 0.05 ANOVA).

**Figure 2:**
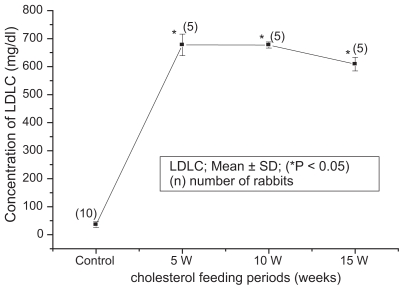
Represents LDLC (mg/dl) concentration in control and cholesterol-fed rabbits versus cholesterol feeding periods of 5, 10 and 15 weeks (*P < 0.05 ANOVA).

**Figure 3:**
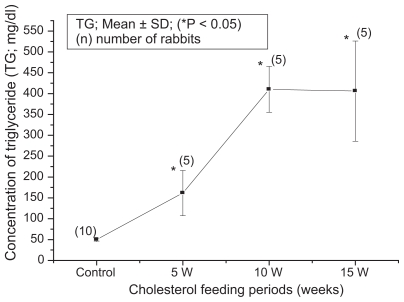
Represents TG (mg/dl) concentration in control and cholesterol-fed rabbits versus cholesterol feeding periods of 5, 10 and 15 weeks (*P < 0.05 ANOVA).

**Figure 4:**
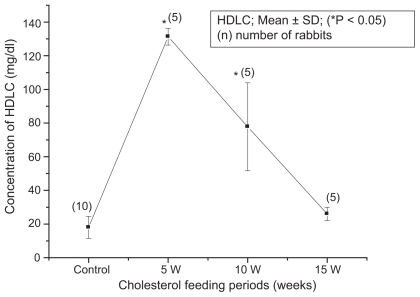
Represents HDLC (mg/dl) concentration in control and cholesterol-fed rabbits versus cholesterol feeding periods of 5, 10 and 15 weeks (*P < 0.05 ANOVA).

**Figure 5:**
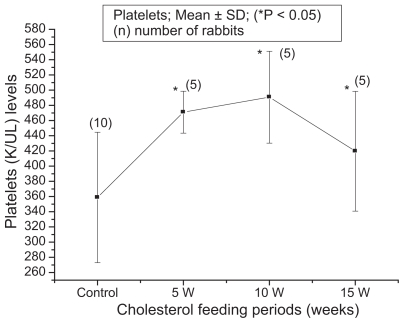
Represents platelets (K/UL) count in control and cholesterol-fed rabbits versus cholesterol feeding periods of 5, 10 and 15 weeks (*P < 0.05 ANOVA).

The blood biochemical data of control and cholesterol-fed group of rabbits were summarized in Figures 1, 2, 3, 4 and 5. Figures 1, 2, 3, 4 and 5 represent TC (mg/dl), LDLC (mg/dl), TG (mg/dl) concentration, HDLC (mg/dl) and platelet counts (K/UL) in control and cholesterol-fed group of rabbits versus cholesterol feeding periods of 5, 10 and 15 weeks. In Figures 1, 2, 3 and 5, TC, LDLC, TG levels and platelet counts were significantly (P < 0.01) increased in cholesterol-fed group of rabbits as compared with control rabbits. In Figure 4, HDLC concentration was significantly (P < 0.01) increased in cholesterol-fed group of rabbits at the feeding periods of 5 and 10 weeks as compared with control rabbits.

Table 1 shows the concentration of different Hyp fractions in control and rabbits fed on high cholesterol diet. The serum Hyp in rabbits belonging to group 1 showed no significant (P > 0.05) alteration in Hyp concentration when compared to control group (unpublished data). Group 2 rabbits showed a significant increase of 103% (P < 0.01) and 100% (P < 0.001) in free and protein—bound Hyp fractions respectively when compared to control rabbits. However, there was no significant change in peptide—bound and total serum Hyp levels as compared to the control group. There was no significant (P > 0.05) decrease of free serum Hyp in group 3 rabbits when compared to control rabbits. On the other hand, group 3 rabbits showed a significant increase in peptide—bound and protein bound Hyp by 517% (P < 0.05) and 100% (P < 0.01) respectively when compared to control rabbits. However, total serum Hyp in group 3 rabbits showed no significant (P > 0.05) change when compared to control rabbits.

## Discussion

In the present study, the rabbits were fed a high cholesterol and saturated fat diet containing 1% cholesterol plus 1% olive oil (100 g/day) for feeding periods of 5 weeks (group 1), 10 weeks (group 2) and 15 weeks (group 3). The accompanying changes in TC, LDLC, TG levels, platelets counts and hydroxyproline fractions of rabbit’s serum during these feeding periods were investigated. Judging from the results obtained in the present study, it became evident that in the hypercholesterolemic rabbit model adopted in the present study, cholesterol feeding could help in the diagnosis and monitoring the progression of atherosclerosis. As one of the possible causes for these changes, it is suggested that cholesterol feeding has a general tendency to induce increase in TC, LDLC, TG levels, platelet counts and various Hyp fractions of the rabbit’s serum which may be deposited in the atheromatous lesions. In fact, the increase of TC, LDLC and TG concentrations has been reported in earlier studies (Gregory, 1999; Sloop, 1996). A high-cholesterol diet elevated level of plasma TC and LDLC which may be incorporated into atherosclerotic plaques. Moreover, as another possible cause, it is suggested that high-cholesterol diet may accelerate atherogenesis through increasing blood viscosity and disturbing the mechanical fragility of atherosclerotic plaques making them vulnerable to rupture and thrombosis. Low HDLC levels are associated with an elevated blood viscosity, and this rheological abnormality contributes to cardiovascular risk (Thomas et al. 1999). Serum LDL and HDL cholesterol levels have effects on blood viscosity and correlate with increased risk of atherogenesis (Sloop and Garber, 1997). Isolated chylomicrons, VLDLC, and LDLC added to plasma or serum in vitro cause a dose-dependent and exponential rise in viscosity (Seplowitz et al. 1981). Hypercholesterolemia modulates haemostasis by altering the expression and/or function of thrombotic, fibrinolytic and rheologic factors (Rosenson et al. 1998). Finally, it is suggested that when LDLC is oxidized by macrophages in lesions, it becomes toxic to the endothelium, and thereby could injure endothelial cells. Thus, the effects of high cholesterol diet are not only confined to deposition of lipids in the atheromatous lesions, but it may produce primary endothelial injury. In the present study, the increase of platelet counts in the experimental rabbits is attributed to high cholesterol diet which promotes platelet aggregation through making them more sensitive to aggregatory agents. In vitro studies, it has been reported that hypercholesterolemic patients were more sensitive to aggregatory agents than platelets from normocholesterolemic (Corash et al. 1981; Di et al. 1986). It has been reported that LDLC binds directly to platelet glycoprotein receptor which serves as the binding site for several agonists including fibrinogen, fibronectin, thrombospondin and vitronectin (Hantgan et al. 1990; Asch et al. 1990; Plow et al. 1985; Parise et al. 1986). Collagen is abundantly found in the connective tissues and serves to provide mechanical strength and support to the tissues. About 13% of the amino acid content of collagen is hydroxyproline which is formed post translationally by hydroxylation of proline. The release of collagen breakdown products in the body fluids like serum and urine has been used as an indicator of collagen breakdown (Siiddiqi and Alhomida, 2005; Siiddiqi and Alhomida, 2006). The hydroxyproline content in the serum and plasma vary in different species indicating the difference in metabolism of collagen in different species (Siiddiqi et al. 2002; Siiddiqi and Alhomida, 2002). Abnormalities in lipid metabolism appear to play a pathogenic role in progressive renal and cardiac diseases. Studies of Rekhter et al., 2000 have demonstrated that hyper-cholesterolemia renders atherosclerotic plaques prone to rupture. This is due to the fact that hypercholesterolemia is accompanied by collagen degradation in the plaque and thereby weakening the plaque (Rekhter et al. 2000). In the present study, feeding high cholesterol and saturated fat diet to rabbits for 10 weeks caused a significant increase in free Hyp in the serum. The pool of free Hyp has a complex origin (Adams and Frank, 1980). It can arise from mature collagen, newly synthesized collagen, from dietary collagen or from propeptides of collagen. Other animal proteins like elastin also contribute to free Hyp pool (Bentley and Hanson, 1969). One or more of these processes could have contributed to the high free serum Hyp after feeding high cholesterol and saturated fat diet to rabbits. However, continued feeding of high cholesterol and saturated fat diet for 15 weeks caused a decrease in free serum Hyp levels which may be a consequence of fall in serum total cholesterol, LDLC and HDLC in the serum. Feeding rabbits with high cholesterol and saturated fat diet caused a significant increase in protein—bound Hyp in the serum. The protein—bound Hyp was not detectable in the control rabbits. In our earlier studies, we had reported the presence of all the three forms of Hyp in rabbit plasma (Siiddiqi et al. 2002). The protein—bound Hyp is also called collagen like protein or hypoprotein. Studies of Nagelschmidt and Struck, 1977 have suggested that protein bound hydroxyproline does not mirror collagen turnover but may be more relevant to C1q or complement metabolism. In the present study, feeding rabbits diet rich in high cholesterol and saturated fat caused no significant change in the total serum Hyp. These results suggest that high cholesterol and saturated fat diet causes changes in various Hyp fractions in serum without any significant change in the total Hyp content. In conclusion, the present results strongly suggest that feeding on rabbits a high cholesterol and saturated fat diet for feeding periods of 5, 10 and 15 weeks significantly increased TC, LDLC, TG, platelets and Hyp fractions in rabbit’s serum. Further studies are required to investigate whether hypercholesterolemia lowering therapies may improve impaired blood biochemistry in rabbits and prevent cardiovascular disorders.

## Figures and Tables

**Table 1:** Changes in various hydroxyproline fractions in the serum of rabbits fed on high cholesterol diet (Mean ± SD).

Group	Free Hydroxyproline μg/ml	Peptide—bound Hydroxyproline mg/ml	Protein—bound Hydroxyproline mg/ml	Total Hydroxyproline mg/ml
Control	26.27 ± 5.66	27.16 ± 2.89	ND	156.33 ± 22.40
Group 2	53.35 ± 11.72**	68.19 ± 8.79^ns^	139.51 ± 32.12***	242.45 ± 61.76^ns^
Group 3	16.19 ± 1.92^ns^	167.92 ± 72.30*	95.57 ± 16.33**	279.69 ± 68.67^ns^

* P < 0.05 as compared to control group;

** P < 0.01 as compared to control group;

*** P < 0. 001 as compared to control group (Tukey’s multiple comparison test). ND—non detectable.

ns Nonsignificant as compared to control group. Group 2 (rabbits fed on high cholesterol diet for 10 weeks), Group 3 (rabbits fed on high cholesterol diet for 15 weeks).

## Abbreviations

TC: Total cholesterol
LDLC: low-density lipoprotein cholesterol
HDLC: high density lipoprotein
VLDLC: very low-density lipoprotein cholesterol
TG: triglyceride
Hyp: hydroxyproline
